# Internet usage and environmental governance satisfaction in China: environmental pollution perception as a mediator

**DOI:** 10.3389/fpubh.2025.1520675

**Published:** 2025-02-03

**Authors:** Xiaorui Huang

**Affiliations:** College of International Relations, Huaqiao University, Xiamen, China

**Keywords:** environmental governance, environmental pollution perception, expectancy-disconfirmation theory, internet usage, mediation analysis

## Abstract

**Background:**

Public perception and satisfaction with environmental governance are essential for evaluating the effectiveness of national environmental policies and advancing the United Nations Sustainable Development Goals (UN-SDGs). However, the role of Internet usage in shaping these perceptions and satisfaction levels remains underexplored. This study examines the influence of different types of Internet use on residents' satisfaction with local environmental governance, with a particular focus on the mediating role of perceived environmental pollution.

**Methods:**

Data were retrieved from 3,046 respondents who participated in the 2021 Chinese Social Survey (CSS). Ordinary least squares (OLS) regression and mediation effect models were employed to analyze the relationships between Internet use, perceived environmental pollution, and satisfaction with environmental governance.

**Results:**

Frequent Internet use for browsing news (β = 0.019, SE = 0.006) and studying (β = 0.020, SE = 0.006) is positively associated with greater satisfaction with environmental governance. However, environmental pollution perception functions as a suppressing mediator in the association of Internet use for news browsing (effect = −0.004, SE = 0.001) and studying (effect = −0.004, SE = 0.001), with environmental governance satisfaction (EGS).

**Conclusions:**

This study is the first to apply expectancy-disconfirmation theory to explore public satisfaction with environmental governance. The findings provide novel insights into the role of Internet usage in shaping perceptions of environmental management and offer practical recommendation for leveraging digital engagement to enhance EGS among the public.

## 1 Introduction

The United Nations (UN) initiated 17 Sustainable Development Goals (SDGs), including clean water and sanitation and affordable clean energy, to foster global partnerships for a sustainable future (1). As a leading developing country, China actively supports the SDGs, making advances in environmental governance. However, its rapid, industry-driven economic growth has led to mounting environmental challenges, such as resource depletion and pollution (2). In 2024, China ranked 154th out of 180 countries in the Global Environmental Performance Index (3). Often, environmental pollution not only hampers China's socioeconomic development but also threatens public health, with ~2 million deaths annually attributed to air pollution (4). In response, the Chinese government has prioritized environmental governance by revising the Environment Protection Law and the Atmospheric Pollution Prevention and Control Law, and launching initiatives like “Internet plus green ecology” to combat pollution and improve the environmental quality (5). In 2023, public expenditure on energy conservation and environmental protection reached 563.3 billion yuan (6). Despite these efforts, public perception and satisfaction with environmental governance, as a critical indicator for evaluating the effectiveness of the policies (7), remains low (8, 9). This paradox highlights the need to explore the factors shaping public perceptions and satisfaction with environmental governance.

In recent days, the Internet, especially following the implementation of “Internet plus green ecology,” has become a predominant tool for environmental governance and a platform for the public to seek and share information on environmental protection (10, 11). This trend raises a critical question for researchers and practitioners: Can Internet use enhance the environmental governance satisfaction (EGS) in the digital era? Existing literature provides contrasting insights (12–14). On one hand, information available on the internet often highlights public policies and efforts related to environmental governance, raising public awareness and fostering satisfaction of government actions (15). On the other hand, new media, including the Internet, might amplify government shortcomings, potentially undermining public trust and satisfaction (16), and negatively affecting evaluations of environmental governance (17). Sometimes, the relationship between Internet use and EGS outcomes could vary across different types of Internet use (18), as previous research suggests that politically-oriented Internet use was significantly associated with individuals' satisfaction on public governance, but entertainment-oriented internet use showed no significant correlation (19). With Internet penetration in China reaching 77.5% and over 1.09 billion users engaging in more diverse internet activities (20), understating whether and how Internet usage contribute to environmental governance satisfaction might be critical for advancing sustainable development practices.

Moreover, environmental pollution perception (EPP) may mediate the relationship between Internet usage and EGS. EPP reflects individuals' subjective assessments of their environment, shaped by both objective observations to personal evaluations (21). Compared with traditional media such as newspaper, the Internet demonstrates a stronger influence on public perceptions and environmental actions (22, 23). Researchers have increasingly examined the impact of Internet use on EPP (24–27), suggesting the Internet provides direct access to environmental information (28), shaping public perceptions (29), yet with contradictory directions. On one hand, online discourse often emphasizes negative environmental scenarios (30), potentially heightening concerns about pollution. Conversely, digital platforms also encourage public engagement with environmental initiatives, fostering a sense of progress and alleviating pollution concerns (31). These varying effects may stem from differences in Internet usage types and individuals' digital literacy. Step forward, negative perceptions of environmental pollutions might significantly decrease satisfaction with environmental governance (25), whereas positive perceptions of environmental quality tend to enhance EGS (32, 33). Despite these insights, the mediating role of EPP in the relationship between Internet use and EGS remains underexplored. Understanding how different types of Internet use shape perceptions of environmental pollution and, in turn, influence governance satisfaction is crucial for advancing sustainable development practices.

## 2 Theoretical framework, hypothesis and aims

**Figure 1:**
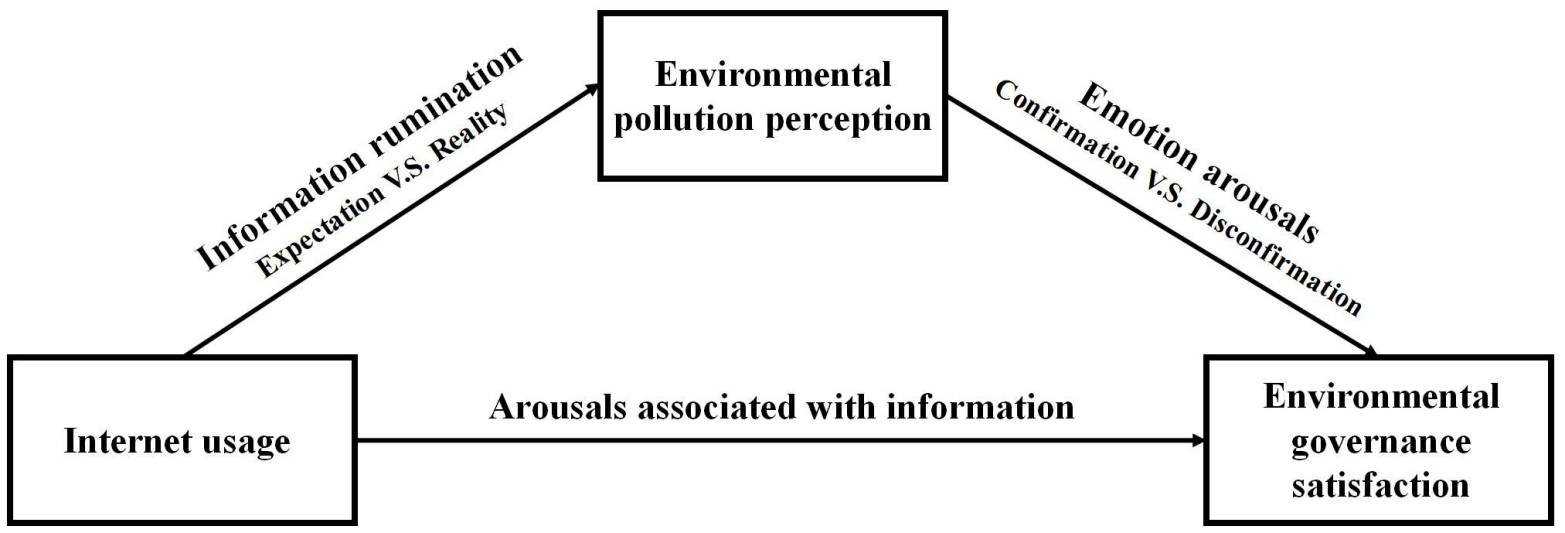
Theoretical framework for the relationship between internet usage, EPP, and EGS.

The relationship between Internet usage, EPP and EGS could be examined through the lens of the expectancy-disconfirmation model (EDM). Originally developed in social and applied psychology to explain consumer satisfaction with products and services (34), the EDM has been adapted over the past two decades to assess the public satisfaction with local governance (35, 36). Accordingly, public satisfaction is derived from information processing comparing perceptions of a public service's performance against prior expectations. More detailed, prior researches suggest that information about past performance significantly shapes citizens' expectations for future outcomes: high past performance leads to high expectations, while low performance lowers them (37). Also, information about environmental protection efforts in other regions may influence the public's expectations of their local government (38). These expectations influence perceptions of current service delivery and satisfaction levels (39). In the context of this study, Internet use enables individuals to access information not only about past performance but also about environmental governance in other regions. This comparative exposure helps shape their perceptions of local environmental pollution and governance efficiencies (40). Subsequently, the alignment or dissonance between these expectations and observed reality impacts overall satisfaction with the government's environmental governance (35). Thus, EPP may mediate the relationship between Internet usage and EGS, as illustrated in Figure 1.

Building on previous studies, we proposed four hypotheses regarding the relationship between Internet usage, environmental pollution perception, and environmental governance satisfaction. The first hypothesis concerns the relationship between Internet use and EGS. We assume two possible outcomes: H1a: Internet use is negatively associated with EGS, as suggested by previous studies (15, 17). Alternatively, H1b: Internet use is positively associated with EGS, in line with prior evidence (41). Given that the impact of Internet use on EGS may vary depending on the type of usage (18), we further propose an exploratory hypothesis: H2: Different types of Internet use have varying impacts on EGS. Additionally, we hypothesize that EPP may mediate the relationship between Internet usage and EGS. Specifically, we propose: H3a: Greater Internet usage may lead to higher levels of EPP, which in turn could decrease EGS, as suggested by previous evidence (30). H3b: Alternatively, increased Internet usage could lower EPP and thus enhance EGS, as indicated by other studies (31). Finally, we propose an exploratory hypothesis, H4: The mediation effect of EPP may differ depending on the type of Internet use.

In summary, this study aims to provide novel insights into the relationship between Internet use and satisfaction with environmental governance, with particular emphasis on the mediating role of environmental pollution perception. These findings aim to deepen the understanding of public satisfaction with environmental protection in the digital era and further extend the applicability of the expectancy-disconfirmation model by offering empirical evidence from the environmental governance context in East Asian.

## 3 Methods

### 3.1 Data sources

This study utilizes data from the 2021 Chinese Social Survey (CSS), a comprehensive, nationwide survey conducted by the Institute of Sociology at the Chinese Academy of Social Sciences. Initiated in 2005, the CSS follows rigorous procedures to ensure the scientific validity and reliability, covering over 150 districts and counties across more than 600 villages and neighborhood committees (42). In the 2021 wave, CSS includes a total of 10,293 respondents from 31 provinces, autonomous regions and municipalities (43). After excluding cases with missing data on Internet usage, environmental pollution perception, and environmental governance satisfaction, the final sample for analysis consisted of 3,046 respondents.

### 3.2 Variable description

Environmental governance satisfaction (EGS), the outcome variable in this study, was measured with the question, “To what extent are you satisfied with governmental environmental protection?” Responses ranged from 1 (very dissatisfied) to 4 (very satisfied), with higher scores indicating greater satisfaction.

Internet usage, the independent variable, was assessed based on the frequency of engaging in different online activities, including browsing current affairs and political news, playing games, chatting and making friends, business or work-related activities, and studying. These categories were adopted and generalized from prior studies (44).

Environmental pollution perception (EPP), the mediating variable, was measured using three questions: “How serious do you think air pollution/water pollution/noise pollution is where you live?” Responses for each question ranged from 1 (“not serious at all”) to 4 (“very serious”). The scores were summed to indicate a composite EPP score ranging from 3 to 12, with higher scores reflecting more severe perceptions of pollution.

Covariates in this study included age, gender, party affiliation, marital status, socioeconomic status (SES), environmental safety, and environmental quality perception (EQP), as these factors have been associated with satisfaction with environmental governance in previous studies (7, 45).

Detailed descriptions of the variables used in this study are presented in Table 1.

**Table 1:** Variable description.

**Variable**	**Variable definition**	**Processing and assignment**	**Min**.	**Max**.
**The dependent variable**				
Environmental governance satisfaction	To what extent do you feel satisfied with governmental environmental protection?	Take the value 1–4, with higher scores indicating greater satisfaction	1	4
**The independent variables**				
**Internet use**				
(a) Browsing news	What's the frequency of use of different online functions?	Take the value 1–6, ranging from 1 (“never”) to 6 (“nearly everyday”)	1	6
(b) Playing			1	6
(c) Chatting			1	6
(d) Business			1	6
(e) Studying			1	6
**Intermediate variable**				
Environmental pollution perception	What do you think of the seriousness of air pollution/water pollution/ noise pollution in the place where you live?	Take the value 1–4, with higher scores indicating greater serious. The scores for each question were summed and used to indicate the PEP, with scores ranging from 3 to 12	3	12
**Control variables**				
Age	What is your date of birth?	2021 minus date of birth to get age	18	70
Gender	What is your gender?	Male = 1, female = 0	0	1
Party	What is your political affiliation?	Member of the Communist Party of China = 1, else = 0	0	1
Marriage	What is your marital status?	Unmarried = 1, married = 2, divorced or widowed = 3	1	3
SES	What level of social and economic status do you think you currently belong to locally?	Take the value 1–5, ranging from 1 (“low level”) to 5 (“high level”)	1	5
Safety	How safe are you in terms of the environment in your current society?	Take the value 1–4, with higher scores indicating greater safety	1	4
Environmental quality perception	What do you think is the state of our country's ecological environment in the world?	Take the value 1–5, ranging from 1 (“low level”) to 5 (“high level”)	1	5

### 3.3. Model specification

Given that the dependent variable in this study is ordinal, ranging from 1 to 4, ordinary least squares (OLS) regression is employed for the analysis. The basic estimation model is specified as follows:


(1) $$\begin{array}{l} \text{EGS }=\;{\text{a}}_{0}+{\text{a}}_{1}\text{Internet}+{\text{a}}_{2}{\text{X}}_{\text{ij}}+\in_{\text{i}} \end{array}$$

In Equation 1, *X*_*ij*_ represents a set of control variables. *a*_0_ is the constant term, *a*_1_, *a*_2_ are regression coefficients, and ∈_*i*_ represents the estimation error.

To test the mediating role of EPP, we follow the approach proposed by Baron and Kenny (46). The mediation mechanism is examined through the following models:


(2) $$\begin{array}{l} \text{EPP }=\;{\text{c}}_{0}+{\text{c}}_{1}\text{Internet}+{\text{c}}_{2}{\text{X}}_{\text{ij}}+\in_{\text{i}} \end{array}$$

(3) $$\text{EGS}={\text{a}}_{0}^{'}+{\text{a}}_{1}^{'}\text{Internet}+{\text{a}}_{2}^{'}\text{EPP}+{\text{a}}_{3}^{'}{\text{X}}_{\text{ij}}+\in_{\text{i}}$$

In Equations 2, 3, *c*_1_ represents the effect of Internet usage on EPP, and $a_{1}^{'}$ represents the direct effect of Internet usage on EGS. $a_{2}^{'}$ represents the effect of EPP on EGS, and *X*_*ij*_ includes the control variables in the mediation models. ∈_*i*_ denotes the error term.

To further evaluate the mediating role of EPP in the relationship between internet use and EGS, bootstrapping methods were employed to obtain point estimates of the coefficients, following the procedures outlined by Preacher and Hayes (47). The bootstrap mediation tests were performed using the sgmediation command in Stata. Lastly, we conducted a robustness test by substituting the OLS model with an ordered probit model.

All methods adhered to relevant guidelines and regulations. Ethical review and approval were waived for this study, as we used publicly available CSS database, which contains anonymized data and involves no experimental procedures posing potential risks to participants.

## 4 Results

### 4.1 Descriptive analysis

As shown in Table 2, among the 3,046 respondents, 44.09% were male, and the average age was 41.38 years (SD = 13.65). Additionally, 53.09% of respondents perceived their socioeconomic status (SES) as above average. In 2021, most residents reported frequent Internet use, engaging in activities such as playing games, chatting, and browsing news multiple times per week. The average score for EPP was 6.05, reflecting a moderate level of concern toward local pollution. Respondents' satisfaction with environmental governance was relatively high, with a mean score of 2.97 (SD = 0.68).

**Table 2:** Demographic characteristics of samples.

**Variable**	**Mean**	**Standard deviation**
**The dependent variable**		
Environmental governance satisfaction	2.97	0.68
**The independent variables**		
Internet use
(a) Browsing news	4.46	1.77
(b) Playing	4.91	1.59
(c) Chatting	4.85	1.67
(d) Business	2.49	2.13
(e) Studying	3.15	2.05
**Intermediate variable**		
Environmental pollution perception	6.05	1.97
**Control variables**		
Age	41.38	13.65
Gender	0.44	0.50
Politics status	0.13	0.34
Marital status	1.99	0.31
SES	3.66	0.89
Safety	3.08	0.60
Environmental quality perception	3.83	0.88

### 4.2 Multivariate regressions

The multicollinearity test revealed that the highest variance inflation factor (VIF) among the variables was 1.14, indicating that multicollinearity was not a concern and the regression assumptions were satisfied. More detailed, Internet usage behaviors such as browsing news (β = 0.019, SE = 0.006) and studying (β = 0.020, SE = 0.006) exhibited signification associations with the outcome variable, EGS. Additionally, EPP demonstrated a robust significant relationship with EGS (beta = −0.090, SE = 0.006). Among the covariates, environmental safety perception (β = 0.35, SE = 0.02) and environmental quality perception (β = 0.13, SE = 0.01) were also significantly associated with EGS. Further details are provided in Table 3.

**Table 3:** Multivariate linear regression analysis of environmental governance satisfaction on different types of Internet use and EPP.

**Variables**	**Model (1)**	**Model (2)**	**Model (3)**	**Model (4)**	**Model (5)**
**Internet use**					
(a) Browsing news	0.019^**^				
	(0.006)				
(b) Playing		0.012			
		(0.007)			
(c) Chatting			−0.005		
			(0.007)		
(d) Business				0.003	
				(0.005)	
(e) Study					0.020^***^
					(0.006)
EPP	−0.090^***^	−0.089^***^	−0.089^***^	−0.085^***^	−0.090^***^
	(0.006)	(0.006)	(0.006)	(0.007)	(0.006)
Age	0.001	0.001	0.000	0.002^*^	0.002^+^
	(0.001)	(0.001)	(0.001)	(0.001)	(0.001)
Gender	−0.062^**^	−0.047^*^	−0.049^*^	−0.019	−0.046^*^
	(0.022)	(0.021)	(0.021)	(0.024)	(0.021)
Politics status	0.068^*^	0.088^**^	0.086^**^	0.102^**^	0.053
	(0.032)	(0.031)	(0.031)	(0.037)	(0.033)
Marital status	0.078^*^	0.077^*^	0.076^*^	0.055	0.078^*^
	(0.033)	(0.033)	(0.033)	(0.038)	(0.033)
SES	−0.021^+^	−0.023^*^	−0.025^*^	−0.063^***^	−0.021^+^
	(0.012)	(0.012)	(0.012)	(0.013)	(0.012)
Safety	0.345^***^	0.344^***^	0.343^***^	0.343^***^	0.343^***^
	(0.019)	(0.019)	(0.019)	(0.019)	(0.019)
EQP	0.134^***^	0.136^***^	0.136^***^	0.135^***^	0.136^***^
	(0.013)	(0.013)	(0.013)	(0.013)	(0.013)
_cons	1.775^***^	1.783^***^	1.900^***^	1.853^***^	1.749^***^
	(0.125)	(0.130)	(0.130)	(0.124)	(0.126)
*N*	3,046	3,046	3,046	3,046	3,046
*R* ^2^	0.308	0.307	0.306	0.306	0.309

EPP, environmental pollution perception; EQP, environmental quality perception.

Standard errors in parentheses, ^+^p < 0.10, *p < , **p < 0.01, ***p < 0.001.

### 4.3 Mediation analysis

Table 4 examines the mediating effect of perceived environmental pollution on the relationship between Internet usage and satisfaction with environmental governance. Based on prior regression analyses, two specific online activities were analyzed: browsing news and studying. Panel A focuses on the activity of browsing news. The results indicate that more frequent online news browsing has a significant positive effect on EPP (β = 0.042, *p* < 0.05). In contrast, heightened perceptions of environmental pollution are significantly associated with decreased satisfaction with environmental governance (β = −0.090, *p* < 0.01). These findings suggest that EPP exerts a suppressing effect on the relationship between Internet use for browsing news and satisfaction with environmental governance. Panel B examines the impact of Internet use for studying. Model 5 shows that Internet use for studying has a significant positive effect on EPP (β = 0.039, *p* < 0.05). Model 6 reveals that EPP significantly reduces satisfaction with governmental environmental protection (β = −0.089, *p* < 0.001). Thus, EPP acts as a suppressing mediator in the relationship between Internet use for studying and satisfaction with environmental governance.

**Table 4:** Test of the mediating effect.

**Panel A: Testing the mediating effects of EPP between Internet use of browsing news and EGS**												
**Variables**	**Outcome: EPP (1)**			**Outcome: EGS (2)**			**Outcome: EGS (3)**			**Outcome: EGS (4)**		
	***beta***	***t***	***p***	***beta***	***t***	***p***	***beta***	***t***	***p***	***beta***	***t***	***p***
Browsing news	0.042	2.03	0.043				0.015	2.23	0.026	0.019	2.94	0.003
PEP				−0.089	−13.28	0.000				−0.090	−13.38	0.000
Age	−0.005	−2.20	0.028	0.000	0.53	0.594	0.001	1.32	0.187	0.001	0.73	0.465
Gender	−0.174	−2.52	0.012	−0.048	−2.27	0.023	−0.047	−2.06	0.040	−0.062	2.16	0.030
Politics Status	−0.102	−1.04	0.297	0.085	2.74	0.006	0.077	2.37	0.018	0.068	2.16	0.030
Marital Status	−0.014	−0.12	0.902	0.077	2.18	0.029	0.079	2.11	0.035	0.078	2.21	0.027
SES	0.105	2.55	0.011	−0.025	−1.99	0.047	−0.030	−2.29	0.022	−0.021	−1.68	0.094
Safety	−0.844	−12.12	0.000	0.343	14.04	0.000	0.420	17.03	0.000	0.345	14.13	0.000
EQP	−0.397	−8.86	0.000	0.135	9.39	0.000	0.170	11.28	0.000	0.134	9.33	0.000
*R* ^2^	0.1416^***^			0.3063^***^			0.2511^***^			0.3084^***^		
*F*	54.01			129.34			99.36			116.39		
**Panel B: Testing the mediating effects of EPP between Internet use of studying and EGS**												
**Variables**	**Outcome: EPP (5)**			**Outcome: EGS (6)**			**Outcome: EGS (7)**			**Outcome: EGS (8)**		
	***Beta***	***t***	***p***	***Beta***	***t***	***p***	***Beta***	***t***	***p***	***Beta***	***t***	***p***
Studying	0.039	2.10	0.036				0.016	2.68	0.007	0.020	3.44	0.001
PEP				−0.089	−13.28	0.000				−0.090	−13.36	0.000
Age	−0.004	−1.29	0.195	0.000	0.53	0.594	0.002	2.17	0.030	0.002	1.89	0.059
Gender	−0.004	−2.04	0.041	−0.048	−2.27	0.023	−0.034	−1.52	0.129	−0.046	2.16	0.031
Politics Status	−0.128	−1.24	0.214	0.085	2.74	0.006	0.064	1.90	0.058	0.053	1.62	0.105
Marital Status	−0.013	−0.11	0.909	0.077	2.18	0.029	0.079	2.13	0.033	0.078	2.23	0.026
SES	0.105	2.56	0.011	−0.025	−1.99	0.047	−0.030	−2.28	0.022	−0.021	−1.66	0.098
Safety	−0.849	−12.16	0.000	0.343	14.04	0.000	0.419	16.98	0.000	0.343	14.05	0.000
EQP	−0.394	−8.79	0.000	0.135	9.39	0.000	0.171	11.35	0.000	0.136	9.43	0.000
*R* ^2^	0.1416^***^			0.3063^***^			0.2516^***^			0.3090^***^		
*F*	53.89			129.34			99.68			116.87		
**Panel C: Testing the mediating effects of EPP in the relationship between Internet usage and EGS (bootstrap** = **5,000)**												
**Mediator**	**Independent variable**			**Conditional indirect effect**			**Boot SE**	**Direct effect**	**Total effect**	**95% CI (Bias-corrected and accelerated)**		
EPP	Browsing news			−0.004			0.001	0.019	0.015	[−0.0074343, −0.0000752]		
EPP	Studying			−0.004			0.002	0.020	0.016	[−0.006932, −0.0001356]		

EPP, environmental pollution perception; EGS, environmental governance satisfaction.

^+^p < 0.10.

^*^p < 0.05.

^**^p < 0.01.

^***^p < 0.001.

To ensure the robustness of these findings, we employed the bootstrap method with 5,000 bias-corrected samples and 95% confidence intervals to test the mediating effect. Confidence intervals excluding zero indicate statistical significance (48). The results, presented in Panel C of Table 4, confirm that EPP significantly mediates the relationship between Internet use for information-seeking and EGS, with an indirect effect of −0.004 (95% CI: −0.007, −0.001). Similarly, the mediation effect of EPP in the relationship between Internet use for studying and satisfaction with environmental governance is also −0.004 (95% CI: −0.007, −0.001). These results indicate that the mechanism and effect of EPP's medication in the relationship between Internet use and EGS is consistent across different types of Internet use.

### 4.4. Robustness test

To enhance the reliability and credibility of this study, we conducted a robustness check using an alternative estimation method. Specifically, we replaced the OLS model with an ordered probit model to assess public satisfaction with environmental governance. The results of this robustness test are presented in Table 5 and further validate the robustness of our conclusions.

**Table 5:** Robust test.

**Variables**	**Outcome: EPP (9)**			**Outcome: Satisfaction (10)**			**Outcome: EPP (11)**			**Outcome: Satisfaction (12)**		
	***Beta***	***t***	***p***	***Beta***	***t***	***p***	***Beta***	***t***	***p***	***Beta***	***t***	***p***
Browsing news	0.026	2.22	0.027	0.036	2.77	0.006						
Studying							0.022	2.04	0.041	0.038	3.19	0.001
PEP				−0.178	−12.87	0.000	–	–	–	−0.178	−12.82	0.000

## 5 Discussion

This study examines the relationship between different types of Internet use, environmental pollution perception, and satisfaction with environmental governance. The findings indicate that more frequent Internet use, particularly for browsing news and educational purposes, is positively associated with higher satisfaction with environmental governance. However, EPP acts as a suppressing mediator in this relationship, as Internet use amplifies perceptions of environmental pollution, which subsequently reduces satisfaction with local environmental governance. Notably, the association between Internet use and EGS varies across different types of internet activities, yet for those with significant relationships, the mediating role of EPP remains consistent. These nuanced dynamics underscore the complexity of the relationship between Internet use and environmental governance satisfaction and warrant further discussion.

This study reveals that frequent Internet use, particularly for browsing news and studying, positively influences satisfaction with local environmental governance, while activities like gaming, chatting, and business-related use show no significant effects. These differences might stem from their distinct focus of online information gathering and agenda-setting. As previous research has noted, politically-oriented Internet activities, such as browsing news and studying, facilitates access to environmental information and knowledge, thereby having a significant impact on the perception of governance (16). Conversely, entertainment and business-oriented activities often exclude environmental issues from their agenda, limiting their impact on environmental governance satisfaction. Interestingly, contrary to prior suggesting a negative relationship (30), this study finds a positive association between Internet use and satisfaction with environmental governance. This shift may reflect the evolution of online environmental discourse (e.g., the choice environmental issues, the expression of the discourse and the kind of narration), which now emphasizes government achievements rather than shortcomings. Specifically, online environmental-related information provides the public with the flexibility to learn at their own pace and convenience, enabling engagement with a wide range of environmental protection topics (22). This, in turn, not only helps individuals focus on government efforts in environmental protection, but also encourages them to adopt pro-environmental behaviors (49, 50), ultimately fostering a more positive evaluation of governmental actions (51). Given the persistent paradox between improved environmental governance performance and low public satisfaction (16), this study offers timely evidence that increasing internet use provides a valuable opportunity to bridge this gap. Leveraging new media platforms to construct positive narratives about environmental governance could be critical in fostering public satisfaction and advancing environmental policy outcomes.

Furthermore, we propose that environmental pollution perception serves as a suppressing mediator in the relationship between Internet usage, particularly for browsing news and studying, and satisfaction with environmental governance. Without accounting for the heightened EPP, the positive relationship between Internet usage and satisfaction with environmental governance would increase by ~25%. Drawing on the expectancy-disconfirmation model, negative disconfirmation occurs when individuals' perceptions of actual outcomes fall short of their expectations (35). On one hand, the public is exposed to both information about the urgency of environmental protection and negative news regarding local environmental pollution. This dual exposure heightens public awareness of environmental degradation, thereby amplifying EPP (30). On the other hand, Internet use for educational purposes not only increases public awareness of environmental issues but also enhances understanding of governmental actions. This increased awareness may lead individuals to perceive a growing gap between their expectations for governmental responses and the actual environmental actions they observe, intensifying their concerns about environmental pollution (29). As what a significant body of global evidence has shown (52, 53), mass media can strongly influence public views, attitudes, and perceptions of environmental issues, largely through the direct promotion of certain narratives. Such media exposure raises public concerns about local environmental pollution, further contributing to the amplification of EPP. Moreover, the rise in EPP heightens public sensitivity to environmental issues, especially those perceived as unresolved by the government, in line with the negativity bias (54). This bias causes individuals to give disproportionate weight to negative information, such as ongoing environmental problems, reinforcing negative disconfirmation (55). As a result, the psychological gap between individuals' expectations and the reality of governmental actions generates dissatisfaction, ultimately reducing satisfaction with government environmental performance. In light of these dynamics, this study highlights the critical role of addressing pollution concerns to enhance the efficiency of environmental governance. Moreover, the applicability of the expectancy-disconfirmation model in explaining public satisfaction with environmental governance suggests that satisfaction is not solely determined by perceptions of policy effectiveness but is also significantly influenced by individuals' expectations of these policies and practices. Understanding public expectations is, therefore, essential for accurately assessing satisfaction with environmental policies. Additionally, tracking changes in citizens' perceptions of performance, expectations, and satisfaction over time can provide valuable insights for improving environmental governance practices and policies, aligning them more closely with public expectations.

This study is one of the first to examine the relationship between Internet usage, environmental pollution perception, and satisfaction with environmental governance using the expectancy-disconfirmation model. While the findings offer valuable insights, several limitations must be acknowledged. First, the cross-sectional design limits causal inference. Longitudinal research is needed to establish temporal relationships between Internet usage, EPP, and satisfaction. Second, the study assumes that increased Internet usage heightens the gap between expectations and reality regarding environmental pollution, but lacks direct measures of these expectations. Future studies should explore how these expectations and perceptions affect satisfaction with more detailed data. Finally, while the study identifies a general trend, it does not account for variations across different sub-populations (e.g., by socioeconomic status or occupation). Further research should explore these differences to provide a more nuanced understanding. Despite of these limitations, this study unravels the dynamics of Internet usages affects public satisfaction with environmental governance, and shed light on fostering public satisfaction and advancing environmental policy outcomes.

## 6 Conclusion and implications

This study, based on a sample of 3,046 adults from China, is one of the first to examine the mediating role of environmental pollution perception in the relationship between different types of Internet use and satisfaction with governmental environmental governance. The findings show that more frequent Internet use for browsing news and studying is associated with higher satisfaction with governmental environmental initiatives. However, EPP acts as a suppressing mediator in this relationship. Specifically, while Internet use increases EPP by raising awareness of environmental pollution, this heightened awareness, disconfirmed by previously high expectations, reduces satisfaction, thus dampening the positive effect of Internet use on satisfaction with environmental governance.

Based on these findings, several policy implications are proposed. First, optimizing environmental communication strategies on online platforms can be essential. With the growing trend of Internet use, governments could seize the opportunity to improve public satisfaction with environmental governance by emphasizing positive environmental outcomes and countering misinformation via digital platforms. Adopting a positive narrative around environmental pollution and protection could stimulate positive psychological responses and enhance public satisfaction. Second, while online environmental education can raise awareness, it is crucial to enhance transparency and communication to bridge the gaps between public expectations and real-world environmental observations. Tracking changes in the public' perceptions of performance, expectations, and satisfaction over time might be helpful to design and implement more effective environmental governance practices and policies.

## Data Availability

The data analyzed in this study is subject to the following licenses/restrictions. The data analyzed in this study was sourced from the China Social Survey (CSS), which is accessible solely upon submission of an application and subsequent approval from the CSS research center. The following stipulations apply: access to the dataset is restricted to academic research purposes and may necessitate signing a data usage agreement. Requests for access to these datasets should be directed to the CSS Data Service Platform at: http://css.cssn.cn. Requests to access these datasets should be directed at: http://css.cssn.cn.
